# Multiple RNA Processing Defects and Impaired Chloroplast Function in Plants Deficient in the Organellar Protein-Only RNase P Enzyme

**DOI:** 10.1371/journal.pone.0120533

**Published:** 2015-03-20

**Authors:** Wenbin Zhou, Daniel Karcher, Axel Fischer, Eugenia Maximova, Dirk Walther, Ralph Bock

**Affiliations:** Max-Planck-Institut für Molekulare Pflanzenphysiologie, Potsdam-Golm, Germany; Max-Planck-Institute for Terrestrial Microbiology, GERMANY

## Abstract

Transfer RNA (tRNA) precursors undergo endoribonucleolytic processing of their 5’ and 3’ ends. 5’ cleavage of the precursor transcript is performed by ribonuclease P (RNase P). While in most organisms RNase P is a ribonucleoprotein that harbors a catalytically active RNA component, human mitochondria and the chloroplasts (plastids) and mitochondria of seed plants possess protein-only RNase P enzymes (PRORPs). The plant organellar PRORP (PRORP1) has been characterized to some extent *in vitro* and by transient gene silencing, but the molecular, phenotypic and physiological consequences of its down-regulation in stable transgenic plants have not been assessed. Here we have addressed the function of the dually targeted organellar PRORP enzyme *in vivo* by generating stably transformed *Arabidopsis* plants in which expression of the *PRORP1* gene was suppressed by RNA interference (RNAi). *PRORP1* knock-down lines show defects in photosynthesis, while mitochondrial respiration is not appreciably affected. In both plastids and mitochondria, the effects of *PRORP1* knock-down on the processing of individual tRNA species are highly variable. The drastic reduction in the levels of mature plastid tRNA-Phe(GAA) and tRNA-Arg(ACG) suggests that these two tRNA species limit plastid gene expression in the *PRORP1* mutants and, hence, are causally responsible for the mutant phenotype.

## Introduction

In all organisms, transfer RNAs (tRNAs) are synthesized as precursor transcripts that undergo extensive post-transcriptional processing, before they can be aminoacylated and serve as amino acid donors in protein biosynthesis. tRNA maturation involves processing of the 5’ and 3’ ends as well as extensive chemical modification of individual nucleosides [1–4]. Plant cells need tRNA-processing and tRNA-modifying enzymes in three distinct cellular compartments (nucleocytosolic compartment, mitochondria and plastids). In recent years, several enzymes involved in tRNA processing and tRNA modification have been identified for all three compartments (e. g., [5–9]).

In eukaryotes and most prokaryotes, the mature 5’ end of tRNAs is generated by the endoribonuclease RNase P. An exception are some archaea that lack an RNase P activity and synthesize 5’ leaderless tRNAs [10]. In most organisms that possess RNase P, it is a ribonucleoprotein consisting of a highly structured RNA and one or more protein subunits [11, 1]. The catalytic activity resides in the RNA component of RNase P, thus qualifying it as a ribozyme [12–15]. Evidence for the existence of a different type of RNase P that lacks an RNA component was first provided for spinach chloroplasts [16]. However, the subsequent identification of putative RNase P genes in the plastid genomes of algae cast some doubt on the existence of protein-only RNase P enzymes [17–20]. Only recently a protein-only RNase P was unambiguously identified. The enzyme, isolated from human mitochondria, is composed of three protein subunits, called MRPP1, MRPP2 and MRPP3, of which subunit 3 was suggested to be catalytically active [21]. Subsequently, based on sequence similarity to MRPP3, RNA-free RNase P enzymes were also identified from the model plant *Arabidopsis thaliana* [7, 8] and from the moss *Physcomitrella patens* [22]. The *Arabidopsis* nuclear genome contains three putative homologs of MRPP3, termed PRORP1, PRORP2 and PRORP3. PRORP2 and PRORP3 are localized in the nucleus, where they process tRNAs and small nucleolar RNAs (snoRNAs). Lack of mutant phenotypes in *prorp2* and *prorp3* single mutants and embryo lethality of *prorp2/prorp3* double mutants suggests that the two proteins exert overlapping, if not redundant, functions in the nucleocytosolic compartment [8]. By contrast, PRORP1 is targeted to both DNA-containing cell organelles. Evidence from *in vitro* studies [7] and transient transformation experiments (using virus-induced gene silencing; [8]) suggests that both mitochondrial and chloroplast tRNAs (as well as some mitochondrial mRNAs harboring tRNA-like secondary structures at their termini) are substrates of PRORP1. Interestingly, PRORP1 can also rescue an RNase P-deficient *Escherichia coli* strain [7], providing further evidence for the protein being sufficient to faithfully perform tRNA 5’ maturation.

Although the identification of PRORP1 as organellar RNase P is now well established, the phenotypic and physiological consequences of PRORP1 deficiency and the relative importance of RNase P functions in plastids versus mitochondria have not been assessed. This is due to *PRORP1* being an essential gene in *Arabidopsis* that cannot be knocked out [7]. Here we have generated stable transgenic plants, in which *PRORP1* is down-regulated by RNA interference (RNAi). Characterization of the molecular and physiological defects in the mutants revealed that, while there is little evidence for impaired mitochondrial function, photosynthesis as the main function of chloroplasts is severely affected. Moreover, the effects on the processing of different tRNA species in mitochondria and plastids are highly variable. Extremely low levels of mature plastid tRNA-Phe(GAA) and tRNA-Arg(ACG) suggests impaired maturation of these two tRNA species as a main cause of the RNase P-deficient mutant phenotype.

## Materials and Methods

### Plant material and growth conditions

Wild-type and transgenic *Arabidopsis thaliana* lines used in this study all are in the Columbia ecotype background. The TadA knock-out mutant *tada-1* was described previously [6] For plant growth on synthetic medium, surface-sterilized seeds were stratified for 3 days, then sown onto half-strength Murashige and Skoog (MS) medium [23] with 1% sucrose and grown in controlled environment chambers. For phenotypic analyses, plants were transferred to soil 7 days after germination on synthetic medium and grown under long-day conditions (16 h light/8 h dark) at a light intensity of 120 μmol photons m^-2^ s^-1^ at 22°C. For measurements of chlorophyll fluorescence and respiration, plants were grown under short-day conditions (8 h light/16 h dark).

### Generation of *PRORP1* RNAi plants

The transformation vector for down-regulation of *PRORP1* in *Arabidopsis* was obtained from the AGRIKOLA collection of RNAi vectors [24]. Plasmid CATMA2a30510 carries a 402-bp PCR product corresponding to positions 74 to 475 of the *PRORP1* coding region. The gene-specific tag of *PRORP1* (in CATMA2a30510) was recombined from the pENTR207 vector into pK7GWIWG2(I) [25]. The construct was then transformed into *Agrobacterium tumefaciens* strain GV3101 and introduced into *Arabidopsis thaliana* wild-type plants via the floral dip method [26]. Transgenic plants were selected for kanamycin resistance by germination of surface-sterilized seeds on agar-solidified half-strength MS medium containing 1% sucrose and 50 mg L^-1^ kanamycin. Resistant seedlings were transferred to soil and grown to maturity in a controlled environment chamber under long-day conditions.

### Pigment analysis

Chlorophyll and carotenoid contents were measured spectrophotometrically according to published protocols [27]. All measurements were performed with three biological replicates.

### Measurement of respiration activity

Oxygen consumption of *Arabidopsis* leaves was measured in the dark at 25°C using a Clark-type oxygen electrode (Hansatech Instruments). Respiration activity of mutant and wild-type leaves was determined by cutting leaf samples into small pieces with a sharp razor blade and adding leaf pieces equivalent to 15 to 25 mg fresh weight to the measuring chamber containing 1 mL of buffer (10 mM MES-KOH, pH 6.5).

### Analysis of chlorophyll fluorescence

Chlorophyll *a* fluorescence at room temperature was measured in intact plants using a pulse amplitude modulated fluorometer (DUAL-PAM-100; Heinz Walz GmbH; [28]). Plants were dark adapted for 15 min prior to all measurements. The minimum fluorescence in the dark-adapted state (*F*_0_) was excited by a weak measuring light (650 nm; 0.05 to 0.1 μmol photons m^-2^ s^-1^). A saturating pulse of white light (800 ms, 3000 μmol photons m^-2^ s^-1^) was applied to determine the maximum fluorescence in the dark-adapted state (*F*_m_) and during illumination with actinic light (*F*_m_’). The maximum photochemical efficiency of photosystem II (PSII) was calculated as [*F*_v_/*F*_m_ = (*F*_m_-*F*_0_)/*F*_m_]. The steady state fluorescence level (*F*_s_) was recorded during illumination with actinic light (15 to 1000 μmol photons m^-2^ s^-1^). The minimum fluorescence in the light-adapted state (*F*_0_’) was measured in the presence of far-red light after the actinic light had been turned off. The quantum yield of PSII (Ф_PSII_) was calculated as (*F*_m_’- *F*_s_)/*F*_m_’. The relative rate of electron transport through PSII (electron transport rate) was calculated as Ф_PSII_ × light intensity (μmol photons m^-2^ s^-1^). Non-photochemical quenching (qN) was calculated as 1-(*F*_m_’-*F*_0_’)/(*F*_m_-*F*_0_). For measurements of light-response curves, plants were illuminated at the following light intensities: 0, 18, 30, 42, 62, 83, 105, 135, 173, 222, 280, 347, 432, 540, 661 and 813 μmol photons m^-2^ s^-1^.

Chlorophyll *a* fluorescence emission spectra at 77 K were recorded using a Jasco F-6500 fluorometer (Jasco GmbH) and isolated thylakoid samples [29]. Chlorophyll *a* fluorescence was excited at 435 nm wavelength (10 nm spectral bandwidth). Fluorescence emission was determined using a spectral bandwidth of 1 nm at wavelengths of 650 to 800 nm. For thylakoid isolation, leaves from 20-day-old plants were homogenized in a buffer containing 0.33 M sorbitol, 50 mM HEPES (pH 8.0), 1 mM MgCl_2_ and 2 mM Na_2_EDTA. The slurry was filtered through two layers of Miracloth (Calbiochem) and centrifuged at 10,000 x g for 15 min at 4°C. The pellet was resuspended in the same buffer, and the chlorophyll concentration was adjusted to 10 μg/ml.

### Microscopic techniques

Leaf anatomy was analyzed by light microscopy. To this end, tissue samples of approximately 1 mm^3^ were fixed with 4% paraformaldehyde and 0.2% glutaraldehyde in 0.1 M phosphate buffer (pH 7.4), vacuum infiltrated and incubated at 4°C overnight. The samples were then rinsed in the same buffer, dehydrated in an ethanol series (30, 50, 70, 80, 90 and 100%) by incubation for 1 h in each solution, and infiltrated with Technovit 7100 resin (Heraeus Kulzer) for up to 24 h [30]. Following polymerization at room temperature, light microscopic analysis was performed on 5 μm cross-sections cut with a rotary microtome (RM 2265; Leica) and placed on poly-L-lysine-coated glass slides (Sigma). The slides were air-dried at 37°C for 2 h on a heating plate, and then stained with toluidine blue O (0.05% solution). The stained leaf sections were examined with a motorized epi-fluorescence microscope (Olympus BX61) using the cell^P software (Olympus).

For ultrastructural analysis of chloroplasts and mitochondria, leaf samples were fixed in 2.5% glutaraldehyde in 0.2 M sodium cacodylate (pH 7.4) for a minimum of 8 h. Post-fixation with 2% osmium tetroxide was done in the same buffer for 4 h. After rinsing the leaf samples in cacodylate buffer, dehydration and embedding in Spurr’s epoxy resin were carried out following standard protocols. Semithin sections (1–2 μm) were cut with glass knives, and thin sections for silver impregnation were cut with diamond knives. For electron microscopy, thin sections were stained with 2% uranyl acetate and lead citrate and examined in a Zeiss EM 912 Omega transmission electron microscope (Carl Zeiss).

### Isolation of nucleic acids and hybridization procedures

Total plant DNA was extracted from fresh leaf material using a cetyltrimethylammoniumbromide (CTAB)-based protocol [31]. Total RNA was isolated using a guanidine isothiocyanate/phenol-based method (peqGOLD TriFast; Peqlab GmbH) following the manufacturer’s instructions. For northern blot analysis, RNA samples were electrophoretically separated in formaldehyde-containing 1.5% agarose gels and transferred to positively charged nylon membranes (Roche). Hybridizations were performed with digoxigenin (DIG)-labeled probes at 50°C overnight. Probes were produced by labeling PCR products with the PCR DIG Probe Synthesis Kit (Roche). Hybridization signals were detected using the CDP-Star reagent. Pre-hybridization, hybridization, washing and detection were performed according to Roche’s DIG Application Manual. The primers used to generate PCR products for DIG labeling are listed in S1 Table.

### Analysis of tRNA editing and DNA sequencing

The editing status of the plastid tRNA-Arg(ACG) was examined as described previously [5]. Evaluation of sequence data was done by measuring peak heights. The editing efficiency in percent was calculated as the ratio of the peak height of the G signal to the sum of the peak heights of the G and A signals at the editing site (position 34 of the tRNA). The difference in editing efficiency between the wild type and the RNAi-2 line was confirmed in several independent experiments. For DNA sequencing, amplification products were separated by electrophoresis in agarose gels and purified from excised gel slices using a NucleoSpin Extract II kit (Macherey-Nagel).

### Quantitative RT-PCR

Samples of 1 μg total RNA treated with TURBO DNase I (Ambion) was used as a template for first-strand cDNA synthesis in a volume of 20 μl with 1 μl of Superscript III reverse transcriptase (Invitrogen). cDNAs were used as templates for quantitative real-time PCR (qRT-PCR). Amplification reactions were carried out with the StepOnePlus real-time PCR system (Applied Biosystems) using Absolute SYBR Green ROX mix (Thermo Scientific) for quantitation. Three biological replicates were analyzed. The 2^-ΔΔCT^ method was applied to determine relative transcript levels [32]. Reactions for each tested gene in each cDNA sample were independently repeated at least three times. *EF1alpha* (At5g60390) was used as a reference (for primer sequences, see [33]). Specific primers for amplification of *PRORP1* (*At2g32230*) were Pprorp1_5’ (5'-GTTTGATGCAGTCATTGATGGAGC-3') and Pprorp1_3’ (5'-TACACGACTCTTGTGCAGGATCAC-3').

### Protein isolation and immunoblotting

Total cellular protein from *Arabidopsis* plants was extracted from 100 mg tissue ground in liquid nitrogen and suspended in 150 μL extraction buffer (4 mM Tris-HCl, pH 7.5; 5 mM NaCl; 6.25 μM MgCl_2_; 10 μM EGTA; 10 μM DTT; 1% Triton X-100; protease inhibitor cocktail). Cell debris were removed by centrifugation at 12,000 rpm for 20 min at 4°C. The protein concentration was measured with a protein assay kit (Bio-Rad) using a dilution series of bovine serum albumin (BSA) as standard. For western blot analysis, protein samples were separated by electrophoresis in 12% SDS-polyacrylamide gels and blotted onto polyvinylidene difluoride (PVDF) membranes (GE Healthcare). Membranes were treated with blocking buffer (20 mM Tris-HCl, pH 7.6; 137 mM NaCl; 0.5% BSA) for 1 h and then incubated with the appropriate primary antibody. Detection was performed with the ECL Plus system (GE Healthcare).

### tRNA sequencing (tRNAseq) and data analysis

Samples of 12 μg of total RNA (extracted from wild-type plants or RNAi-2 mutant plants) were separated on urea-containing 8% polyacrylamide gels followed by staining with ethidiumbromide. The small RNA fraction (~70 to ~150 nt) was excised from the gel, and the RNA was purified as described previously [5]. RNA libraries were prepared using the NEBNext® Small RNA Library Prep kit (New England Biolabs), and sequencing was performed on an Illumina HiSeq2000 sequencer at the Max Planck Genome Centre, Cologne, Germany. Illumina sequencing of size-fractionated RNAs generated 44,992,406 and 48,108,194 raw reads for the wild type and the RNAi-2 line, respectively. These resulted in 40,150,680 genome-mapped reads with an average length of 80 nt for the wild type, and 41,391,939 genome-mapped reads with an average length of 79 nt for the RNAi-2 line. Of those, 2,151,353 and 3,163,815 reads were mapped to tRNA genes in the wild type and the RNAi-2 line, respectively (S1 Fig.).

Illumina reads were checked for quality with the FastQC program (v0.10.1) (http://www.bioinformatics.babraham.ac.uk/projects/fastqc) after trimming adapter sequences at the 3'-ends with Scythe (v0.991) (https://github.com/vsbuffalo/scythe) using fastqc_contaminant_list.fa provided by FastQC. Trimmed reads were then mapped to unique tRNA sequences of *Arabidopsis thaliana* downloaded from the plant tRNA database (http://plantrna.ibmp.cnrs.fr/) and the *Arabidopsis thaliana* TAIR 10 genome downloaded from EnsemblPlants, release 19 (ftp://ftp.ensemblgenomes.org/pub/release-19/plants/fasta/arabidopsis_thaliana/dna/Arabidopsis_thaliana.TAIR10.19.dna.toplevel.fa.gz) using BWA (v0.7.0-r313; [34]) to determine tRNA accumulation levels and tRNA processing efficiency, respectively. Given the repetitive nature of tRNA sequences across the genome, no uniqueness filter was set in genome mapping. Samtools (v0.1.19) was used to compress, sort and index the alignment SAM output (view-bS, sort, index) and generating mapping statistics (flagstat; [35]).

For tRNA accumulation levels, mapped reads were counted for each unique mature tRNA sequence in order to compute RPKM (reads per kilobase per million reads) values and calculate differences (M) and averages (A) for the MA plot. In the MA plot, the average of both log_2_ expression values [A = {log_2_(RNAi-2) + log_2_(WT)}/2] is plotted against the difference in log_2_ expression values of the two samples [M = (log_2_(RNAi-2)—log_2_(WT)].

To generate comparable read annotation statistics, mapped reads were annotated according to their first assignment to a genomic feature in the following order using intersectBed of the BEDtools package [36]: exons, 5’ and 3’ untranslated regions (‘prime UTRs’), repeats (excluding ‘dust’) and tRNA flanks (±50 bp of mature tRNA locations). The annotation file in GTF format was obtained from the same EnsemblPlants FTP site (ftp://ftp.ensemblgenomes.org/pub/release-19/plants/gtf/arabidopsis_thaliana/Arabidopsis_thaliana.TAIR10.19.gtf.gz) and converted into BED format to be compatible with BEDtools input formats.

To determine tRNA processing efficiency, read counts were estimated by intersecting genome mapped data with mature tRNA regions and tRNA flanks (±50 bp of mature tRNA locations) using intersectBed [36] with-f 0.3 and 0.1, respectively. Afterwards, a processing efficiency rate (PER), defined as the log_2_ ratio of reads mapped on mature and 3' or 5' flanking region, respectively, was computed for each tRNA in the wild type and the RNAi line to calculate differences (M) and averages (A). In the MA plot, the average of both log_2_ expression values [A = {log_2_(RNAi-2) + log_2_(WT)}/2] is plotted against the difference in log_2_ expression values of the two samples [M = (log_2_(RNAi-2)—log_2_(WT)]. The eight plastid tRNAs harboring introns were not considered in the processing efficiency computations, because of the inevitable algorithmic uncertainties associated with gapped transcript read mapping on genome sequences. Additionally, for the five tRNAs without intron that are present as exact forward and inverted duplicate pairs, read counts were combined and assigned to the forward strand instance only. In all subsequent analyses, the respective inverted repeat instance was ignored.

For three tRNAs occurring twice in the mitochondrial genome and which have identical mature tRNA sequences and identical 50 bp flanking sequences (but are not annotated as duplicated regions), read counts were also combined and assigned to the instance listed first on the mitochondrial sequence, as defined by genome coordinates. This instance was then used in all subsequent analyses and all others were ignored.

R v3.0.2 was used for all further statistical analyses and visualizations (http://www.R-project.org/) including P value calculation and adjustment using Fisher’s exact test and Benjamini-Hochberg multiple testing correction, respectively.

## Results and Discussion

### Generation of stable RNAi lines for the organellar RNase P in *Arabidopsis*

The discovery of a protein-only RNase P in human mitochondria [21] triggered searches for homologs that could process plastid tRNAs. This led to the identification of the three putative MRPP3 homologs from the model plant *Arabidopsis* that were reported recently as PRORP1–3 [7, 8]. As the PRORP1 protein localizes to both plastids and mitochondria [7], we decided to generate stable *PRORP1* mutants to be able to determine the effects of impaired organellar RNase P function on plant growth and development.

**Fig 1:**
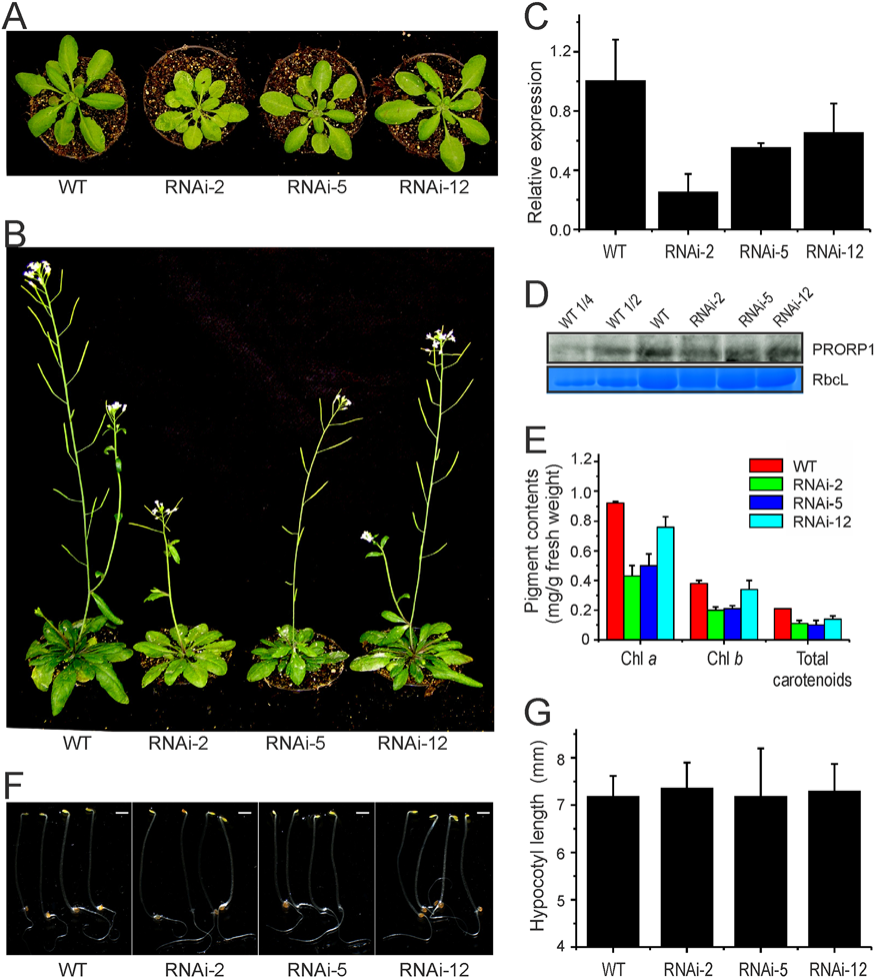
Phenotypic and molecular analysis of *PRORP1* RNAi mutant lines generated in *Arabidopsis*. (**A**) Phenotypes of three independently generated *PRORP1* RNAi mutants (RNAi-2, RNAi-5 and RNAi-12) in comparison to wild-type plants (WT). Seven-day-old seedlings raised on synthetic medium were transferred to soil and grown under long-day conditions for 21 days. (**B**) Phenotypes of the same plants after 35 days under long-day conditions. (**C**) Down-regulation of *PRORP1* expression in the three independently generated RNAi lines as determined by qRT-PCR. Error bars indicate the standard deviation (n = 3). (**D**) PRORP1 protein accumulation in RNAi mutants and wild-type plants. Total protein was extracted from 25 day-old plants grown under long day conditions, and the PRORP1 protein was detected with a specific antibody (kindly provided by Dr. Philippe Giegé). For quantitative assessment of protein accumulation in the RNAi mutants, a dilution series of the wild-type sample (100%, 50% and 25%) was loaded. The Coomassie-stained RbcL protein band is shown as a loading control. (**E**) Pigment accumulation in 20-day-old RNAi mutants and WT plants. Error bars indicate the standard deviation (n = 3). Chl: chlorophyll. (**F**) Phenotypes of five-day-old etiolated seedlings. Scale bar: 1mm. (**G**) Hypocotyl length of 5-day-old etiolated seedlings. Error bars indicate the standard deviation (n = 15).

In the absence of viable T-DNA knock-out lines for *PRORP1* [7], we sought to produce mutant plants by RNA interference (RNAi). Transformation of *Arabidopsis thaliana* plants with a hairpin-type RNAi construct [24] targeted against *PRORP1* yielded several independent transgenic lines, which were subsequently assayed for their phenotypes under standard greenhouse conditions. RNAi is capable of producing a spectrum of phenotypes due to different levels of down-regulation of the target gene in independently generated transgenic lines. Indeed, such a spectrum of phenotypes ranging from a mild reduction in leaf pigment content to severe pigment loss and growth retardation was obtained when *PRORP1* RNAi lines were analyzed (Fig. 1A,B). Three representative RNAi lines (RNAi-2, RNAi-5 and RNAi-12) were selected for in-depth analysis.

Real-time quantitative RT-PCR was employed to measure the level of down-regulation of *PRORP1* expression in the RNAi lines. Consistent with the severity of the phenotypes, RNAi suppression of gene expression was found to be strongest in line RNAi-2, with the residual expression level of *PRORP1* being approximately 25% of the expression in the wild type (Fig. 1C), followed by line RNAi-5 (55% residual expression) and line RNAi-12 (65% residual expression). Western blot analysis using an anti-PRORP1 antibody confirmed that the reduction of gene expression was strongest in line RNAi-2 (Fig. 1D).

To preliminarily assess the effects of *PRORP1* down-regulation on chloroplast and mitochondrial function, we determined photosynthetic pigment contents (Fig. 1E) and assayed seedling growth in the dark (Fig. 1F,G). While photosynthetic pigment content can serve as a proxy of chloroplast function, seedling growth in the dark can provide information about mitochondrial function. This is because, in the dark, growth is entirely dependent upon energy production by respiration, the main function of mitochondria and the function that all mitochondrial genes are directly or indirectly involved in [37, 38]. In line with the pale-green phenotype of the RNAi lines, the contents of photosynthetic pigments (chlorophylls and carotenoids) were significantly reduced. The extent of the reduction correlated with the level of down-regulation of *PRORP1* expression and the severity of the phenotype of the mutants (Fig. 1A-E). In contrast, seedling growth in the dark (measured as hypocotyl length) was not appreciably impaired in any of the RNAi mutants, possibly indicating that mitochondrial function is less affected by the knock-down of *PRORP1* than chloroplast function.

### Altered leaf morphology and organellar ultrastructure in *PRORP1* RNAi mutants

**Fig 2:**
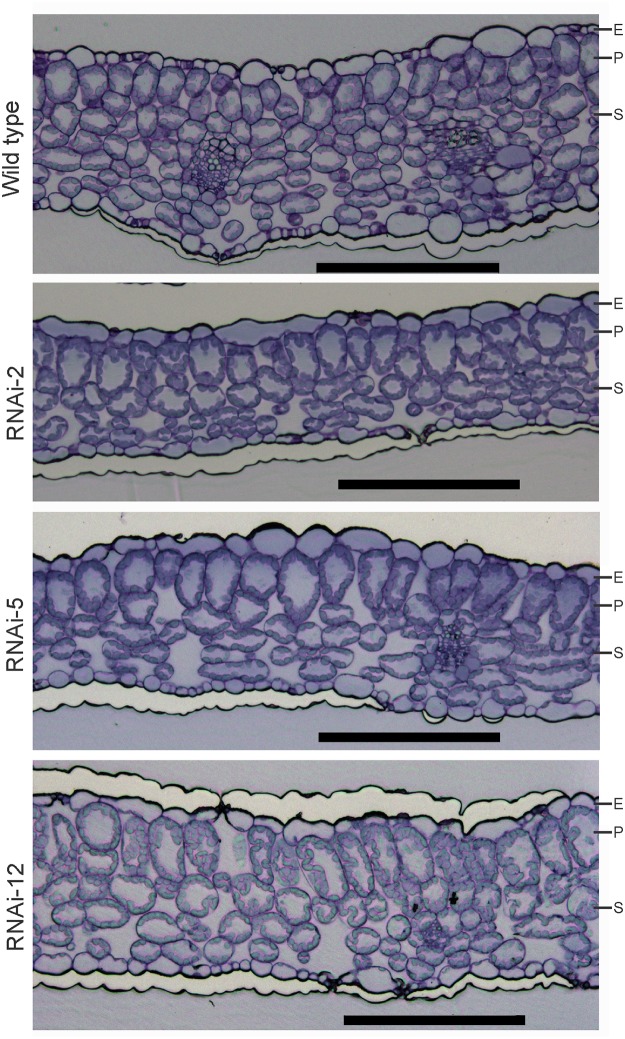
Leaf anatomy in wild-type plants and three independently generated *PRORP1* RNAi mutants (RNAi-2, RNAi-5 and RNAi-12). Cross sections of 15-day-old leaves stained with toluidine blue O are shown. Note reduced spongy mesophyll cell layers and increased size of cylindrical palisade cells in the RNAi plants. E: epidermis; P: palisade parenchyma; S: spongy mesophyll. Scale bars: 200 μm.

In conjunction with the function of RNase P in tRNA processing, the pigment-deficient phenotype of the *PRORP1* RNAi lines provided circumstantial evidence for chloroplast translation being affected in the mutants. The activity of chloroplast gene expression is known to act as the source of a retrograde signal that affects various aspects of plant development, including leaf morphology [39, 40] and anatomy [41, 33]. At the level of leaf anatomy, the most pronounced effects are seen in mesophyll cell proliferation and differentiation. To analyze whether *PRORP1* mutants show defects in mesophyll cell differentiation, we investigated leaf anatomy by epifluorescence microscopy of stained cross sections. Interestingly, the *PRORP1* RNAi lines showed pronounced alterations in mesophyll organization. Compared to wild-type leaves, the number of spongy mesophyll cell layers was reduced in the RNAi plants and the size of cylindrical palisade cells was significantly increased (Fig. 2). Again, the severity of this phenotype at the anatomical level correlated with the severity of the phenotype at the level of the whole plant and with the intensity of *PRORP1* down-regulation (Figs. 1 and 2).

**Fig 3:**
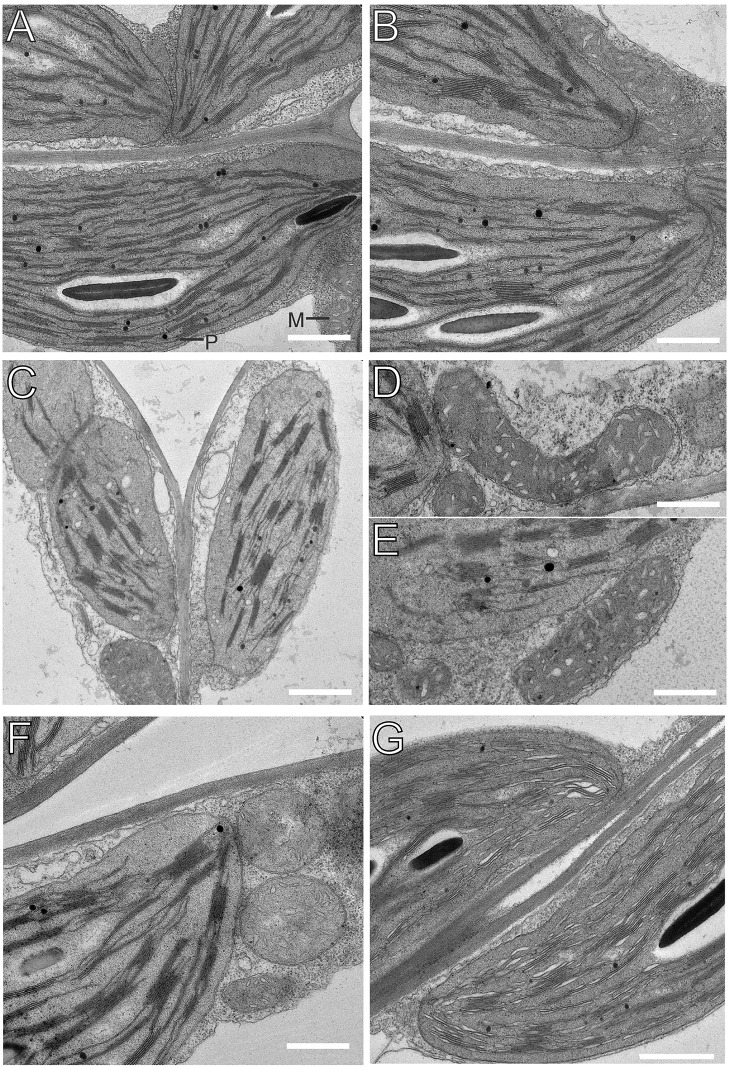
Transmission electron microscopic images of chloroplasts and mitochondria in 15-day-old leaves from *PRORP1* RNAi mutants and wild-type plants. (**A, B**) Ultrastructure of chloroplasts and mitochondria in wild-type cells. For easy organelle identification, a chloroplast (P) and a mitochondrion (M) are labeled. (**C-E**) Ultrastructure of chloroplasts and mitochondria in cells of the strong *PRORP1* RNAi mutant line RNAi-2. Note smaller chloroplasts with more pronounced grana stacking and bigger, more elongated mitochondria. (**F**) Ultrastructure of chloroplasts and mitochondria in line RNAi-5. (**G**) Chloroplast ultrastructure in the weakest RNAi line (RNAi-12). Scale bars: 1 μm.

To assess the consequences of *PRORP1* suppression at the ultrastructural level, chloroplasts and mitochondria were analyzed by transmission electron microscopy (Fig. 3). Conspicuous changes were seen in both chloroplast and mitochondrial ultrastructure, which were most pronounced in the strongest RNAi line, RNAi-2. Chloroplasts were smaller than in wild-type cells and displayed a somewhat more intense stacking of grana thylakoids. This observation is in line with the chlorophyll *a*:*b* ratio being decreased in the mutants (2.43±0.13 in the wild type versus 2.16±0.17 in line RNAi-2; Fig. 1E), which was found to correlate with increased grana stacking also in other mutants with impaired chloroplast function [42]. Mitochondrial shape was also altered in the *PRORP1* RNAi lines. Whereas, in wild-type cells, mitochondria were round or oval, they were bigger and much more elongated in the mutants, especially in cells of the RNAi-2 line (Fig. 3). In contrast to the previously reported transient repression of *PRORP1* (by virus-induced gene silencing; [8]), we did not observe “dense mitochondrial structures containing vacuoles”. Whether this alteration in mitochondrial morphology represents a direct compensatory response to impaired mitochondrial gene expression or rather an indirect consequence of disturbed chloroplast function in the RNAi mutants, remains to be determined.

### Analysis of photosynthesis and respiration in *PRORP1* mutants

**Fig 4:**
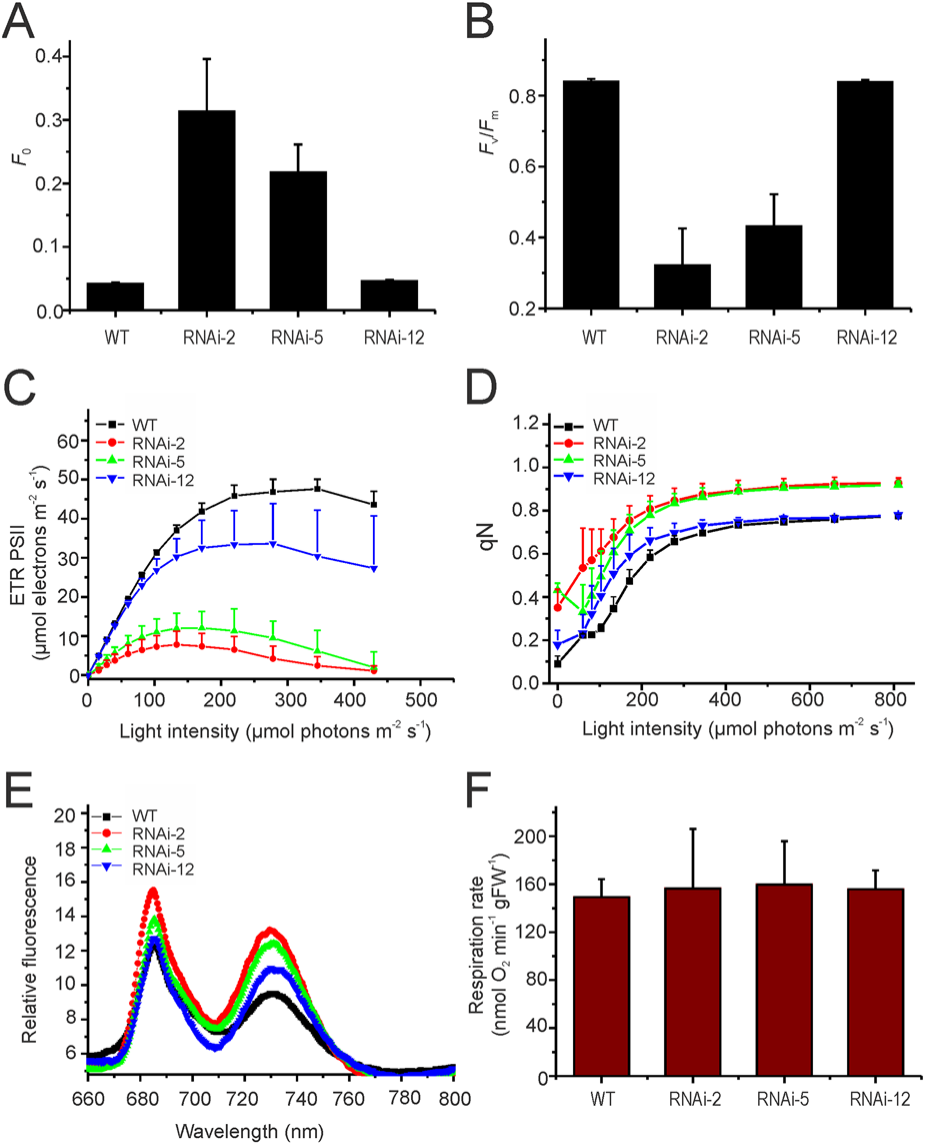
Analysis of photosynthetic activity and mitochondrial respiration in 30-day-old *PRORP1* mutants and wild-type plants grown under short day conditions. (**A**) Minimum fluorescence (*F*_0_). (**B**) Maximum quantum efficiency of PSII (*F*_v_/*F*_m_). (**C**) Light saturation curve of linear electron flux as calculated from the PSII yield. (**D**) Non-photochemical quenching (qN). (**E**) 77K chlorophyll *a* fluorescence emission spectra. Note that the fluorescence emission maxima of PSII (688 nm) and PSI (733 nm in the wild type) are slightly shifted towards shorter wavelengths in the RNAi mutants. (**F**) Measurement of total leaf respiration in the dark (n = 4). FW: fresh weight.

In order to analyze the physiological consequences of knocked-down *PRORP1* expression in more detail, a number of photosynthetic parameters and leaf respiration rates were determined. When photosystem II (PSII) activity was analyzed by measuring chlorophyll fluorescence at room temperature, the RNAi lines showed a strongly increased minimum fluorescence *F*_0_ (Fig. 4A). Elevated *F*_0_ usually results from the presence of free PSII antenna proteins that are uncoupled from PSII reaction centers and is seen both in mutants with deficiencies in PSII core subunits [43] and in mutants with defects in chloroplast gene expression [44,33]. The latter is because most of the reaction center proteins of the photosystems are encoded in the chloroplast genome, whereas all proteins of the light-harvesting antenna are encoded in the nuclear genome. Thus, the enhanced minimum chlorophyll fluorescence emission in the mutants provided circumstantial evidence for a deficiency in PSII reaction centers, which in turn could be caused by a reduced efficiency of chloroplast translation in the absence of sufficient amounts of RNase P for tRNA processing. Consistent with this assumption, the maximum quantum efficiency of PSII (*F*_v_/*F*_m_) was reduced in the mutants (Fig. 4B), with the intensity of the reduction again correlating with the severity of the phenotype and the strength of the RNAi suppression of *PRORP1* expression. Also, the PSII-based electron transport rates were reduced in the mutants in a light intensity-dependent manner (Fig. 4C) and the RNAi plants initiate photoprotective mechanisms (non-photochemical quenching, qN) already in low light (Fig. 4D). Finally, recording of 77K chlorophyll *a* fluorescence emission spectra revealed that the fluorescence emission maxima of both photosystems are shifted towards shorter wavelengths in the RNAi mutants (Fig. 4E), well in line with the presence of antenna complexes that are disconnected from their reaction centers [45, 46].

To assess mitochondrial function, respiration rates in leaves of wild-type plants and *PRORP1* mutant plants were measured (Fig. 4F). Respirational oxygen consumption in mutant leaves was not significantly different from wild-type leaves, indicating that respiration as the main physiological function of mitochondria (and of the gene products encoded in the mitochondrial genome) is not significantly affected by the knock-down of RNase P. Together with the unaltered growth of mutant seedlings in the dark, these results suggest that the mutant phenotype of the *PRORP1* RNAi lines is unlikely to be caused by impaired mitochondrial function, but may be chiefly due to defects in chloroplast gene expression.

### Processing and accumulation levels of organellar tRNAs in *PRORP1* RNAi plants

**Fig 5:**
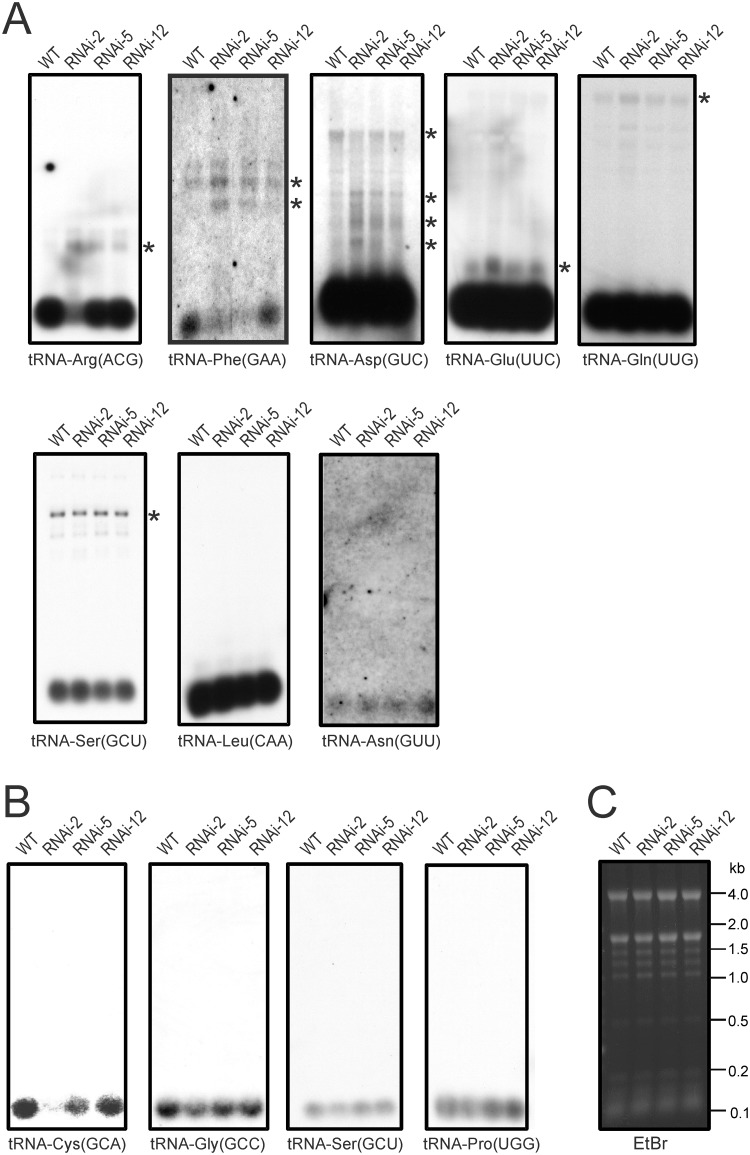
Accumulation and processing patterns of chloroplast and mitochondrial tRNAs in *PRORP1* RNAi mutants and wild-type plants as determined by northern blotting. Detectable precursor RNA species are marked with asterisks. (**A**) Northern blot analysis of chloroplast tRNAs. Weaker exposures of the northern blots for tRNA-Glu(UUC), tRNA-Gln(UUG) and tRNA-Asp(GUC) are presented in S4 Fig. (**B**) Northern blot analysis of mitochondrial tRNAs. (**C**) An ethidium bromide (EtBr)-stained agarose gel photographed prior to blotting as a control for equal loading. Fragment sizes of the RNA size marker are indicated in kilobases (kb).

To directly determine the effects of reduced RNase P function on the maturation and accumulation levels of plastid and mitochondrial tRNAs, a series of northern blot experiments with tRNA-specific probes was performed (Fig. 5). Interestingly, many of the tRNAs showed little, if any, changes in abundance of the mature tRNA species. Most blots needed to be strongly overexposed to visualize the unprocessed precursor RNAs. When this was done (Fig. 5A), some overaccumulation of precursor molecules was detectable in the RNAi lines for many of the plastid tRNAs (which, due to the larger volume of the chloroplast compartment, are generally much more abundant than mitochondrial tRNAs and, therefore, detected at higher sensitivity). As far as the plastid tRNAs are concerned, only two of the investigated tRNA species showed significant reductions in the levels of the mature tRNAs. tRNA-Arg(ACG) was reduced in the strongest RNAi line (RNAi-2; Fig. 5A) and tRNA-Phe(GAA) was reduced to barely detectable levels in the two strongest RNAi lines (RNAi-2 and RNAi-5). In both cases, this reduction in mature tRNA molecules was accompanied by the overaccumulation of precursors (Fig. 5A).

When a set of mitochondrial tRNAs were investigated, a strong reduction was seen for tRNA-Cys(GCA), the mitochondrial tRNA species most intensely investigated in previous studies [7,47]. The other three tRNAs tested showed a somewhat milder reduction in the accumulation of the mature tRNA, which for the tRNA-Ser(GCU) and tRNA-Pro(UGG) was only apparent in the strongest RNAi line (Fig. 5B).

### Mild processing defects in messenger RNAs and ribosomal RNAs in *PRORP1* mutant plants

**Fig 6:**
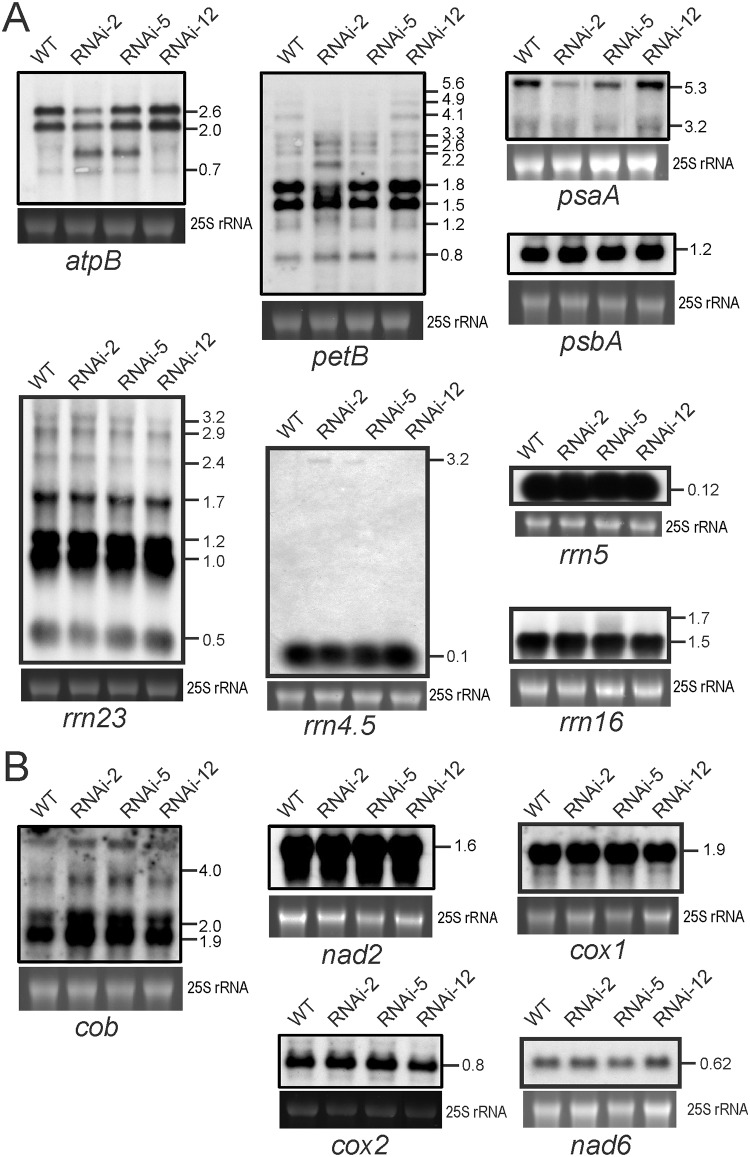
RNA gel blot analyses to assess accumulation and processing of chloroplast and mitochondrial mRNAs and rRNAs in *PRORP1* mutants and wild-type plants. The 25S rRNA band of the ethidium bromide-stained gel prior to blotting is shown as a loading control for all blots. Transcript sizes are indicated in kb. (**A**) Accumulation and processing patterns of chloroplast mRNAs and rRNAs as determined by northern blotting. (**B**) Accumulation and processing patterns of mitochondrial mRNAs. The *cox1* mRNA harbors a tRNA-like structure in its 5’ UTR, a so-called t-element, that potentially could be processed by RNase P [49,7].

To confirm that the phenotypic consequences of knocked-down *PRORP1* expression are mainly due to defective tRNA processing, a set of ribosomal RNAs (rRNAs) and messenger RNAs (mRNAs) in plastids and mitochondria was also investigated by northern blotting (Fig. 6). When plastid rRNA species were analyzed, only minor differences were observed. The 0.5 kb hidden-break product of the 23S rRNA (*rrn23*) was slightly reduced in the strongest RNAi line (RNAi-2) and the 3.2 kb precursor of the 4.5S rRNA (*rrn4*.*5*) accumulated to detectable levels in the RNAi-2 and RNAi-5 lines, but not in the wild type (Fig. 6A).

When the accumulation of plastid mRNAs was analyzed, differences in both mRNA abundance and processing patterns were observed upon comparison of wild-type plants and RNAi lines. The most pronounced alteration was a significant decrease in accumulation of the 5.3 kb tricistronic transcript comprising the genes *psaA*, *psaB* (encoding the two PSI reaction center subunits A and B) and *rps14* (encoding protein S14 of the small ribosomal subunit). Minor changes were seen in the processing patterns of the *atpB* operon (comprising the genes *atpB* and *atpE*, both encoding subunits of the chloroplast ATP synthase) and the pentacistronic transcript of the *psbB* operon (containing three genes for PSII subunits and two genes for subunits of the cytochrome b_6_f complex, *petB* and *petD*; Fig. 6A), whereas no significant changes were seen in *psbA* mRNA accumulation (encoding the D1 reaction center protein of PSII). It is conceivable that the changes in plastid mRNAs contribute to the physiological defects identified in the RNAi mutants (Fig. 4). Whether these alterations in the abundance and processing patterns of non-tRNA transcripts are direct consequences of impaired RNase P function (in that these RNAs are processed by PRORP1) or, alternatively, are secondary consequences of reduced plastid translational activity, is currently unclear. The latter scenario is conceivable, because previous research has established that defective plastid translation causes diverse alterations in plastid RNA metabolism [48]. Importantly, impaired translation does not affect all mRNAs equally. While some chloroplast mRNAs are destabilized as a consequence of their decreased association with ribosomes, others accumulate to normal levels [48].

With the exception of a subtle overaccumulation of the *cob* transcripts (encoding cytochrome b), no significant changes were seen in the accumulation levels and processing patterns of mitochondrial mRNAs. The overaccumulation of *cob*, which affects both the mature mRNA and the unprocessed precursors (Fig. 6B), could be a secondary consequence of a reduced translational activity in the mitochondrion, in that lower coverage with ribosomes results in stabilization of the transcripts. Interestingly, the *cox1* mRNA, which harbors a tRNA-like structure in its 5’ UTR (also referred to as t-element; [49]) was unaffected by the knock-down of RNase P, even though such t-elements have been proposed to be substrates of RNase P [7]. Also, the mitochondrial *nad6* mRNA previously reported to require PRORP1 for its stable accumulation [8] was not significantly reduced in our RNAi mutants (Fig. 6).

### Reduced accumulation of organellar genome-encoded proteins in *PRORP1* mutants

**Fig 7:**
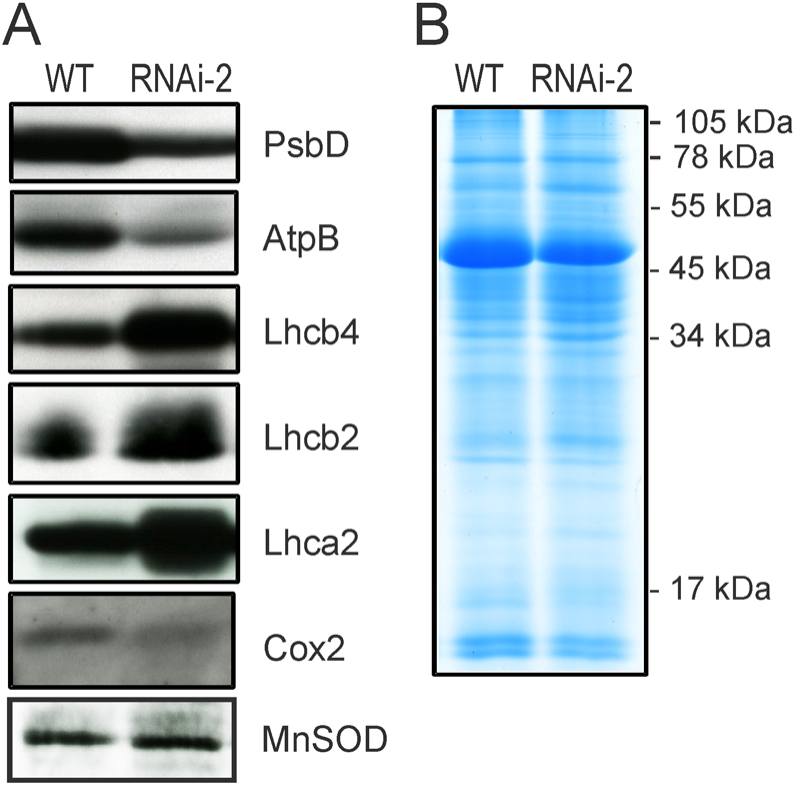
Accumulation of chloroplast and mitochondrial proteins in the wild type and the *PRORP1* mutant line RNAi-2. (**A**) Immunoblot analysis of selected chloroplast and mitochondria proteins. While PsbD and AtpB are chloroplast-encoded proteins, the three light harvesting complex proteins (Lhcb2, Lhcb4 and Lhca2) are nucleus-encoded and post-translationally imported into the chloroplast. Note that the two chloroplast-encoded proteins are strongly reduced in the *PRORP1* mutant, whereas the nucleus-encoded proteins accumulate to higher levels than in the wild type. Cox2, a mitochondrial genome-encoded protein, also accumulates to lower levels in the RNAi-2 mutant plants. Immunoblot analyses were conducted with samples of total cellular protein (20 μg) extracted from leaves and probed with specific antibodies against PsbD (the photosystem II reaction center protein D2), AtpB (the β-subunit of the chloroplast ATP synthase), Lhcb2 and Lhcb4 (light-harvesting proteins of the photosystem II antenna), Lhca2 (a light-harvesting protein of the photosystem I antenna), Cox2 (subunit II of the mitochondrial cytochrome c oxidase), and MnSOD (the nucleus-encoded mitochondrial superoxide dismutase). (**B**) As a control for equal loading, a replicate gel was stained with Coomassie brilliant blue.

To directly confirm that impaired processing of organellar tRNA species results in reduced synthesis of organellar genome-encoded proteins, western blots with specific antibodies against plastid and mitochondrial proteins were performed (Fig. 7). Two plastid-encoded and three nucleus-encoded chloroplast proteins were comparatively analyzed in the wild type and the strongest *PRORP1* RNAi line (RNAi-2): the plastid-encoded PsbD protein (the D2 subunit of the PSI reaction center), the plastid-encoded AtpB protein (the ATP synthase β-subunit) and three nucleus-encoded proteins of the light-harvesting antennae of PSII (Lhcb2 and Lhcb4) and PSI (Lhca2), respectively. Consistent with the *PRORP1* mutants being defective in plastid gene expression, but largely unaffected in nuclear gene expression, accumulation of the plastid-encoded proteins was strongly reduced in the RNAi plants, whereas the nucleus-encoded light-harvesting complex proteins even overaccumulated (Fig. 7A). The latter is probably not due to their overexpression, but rather to the loading of the protein gels based on equal amounts of total cellular protein. As chloroplast-encoded proteins represent a sizeable fraction of the total leaf protein, their reduced accumulation leads to an overrepresentation of nucleus-encoded proteins in the samples.

The limited availability of antibodies against mitochondrial proteins that are sufficiently sensitive to facilitate detection in western blots with total cellular protein allowed only the testing of a single mitochondrial genome-encoded protein, Cox2 (the subunit II of the mitochondrial cytochrome c oxidase). Accumulation of this protein in the *PRORP1* RNAi mutant was significantly reduced, suggesting that impaired processing of mitochondrial tRNAs results in reduced rates of mitochondrial protein biosynthesis. This is a specific effect in that a nucleus-encoded protein analyzed as control (MnSOD) was not reduced in the mutant (Fig. 7).

### Reduced accumulation of plastid tRNA-Arg(ACG) results in lower adenosine-to-inosine editing in *PRORP1* mutants

**Fig 8:**
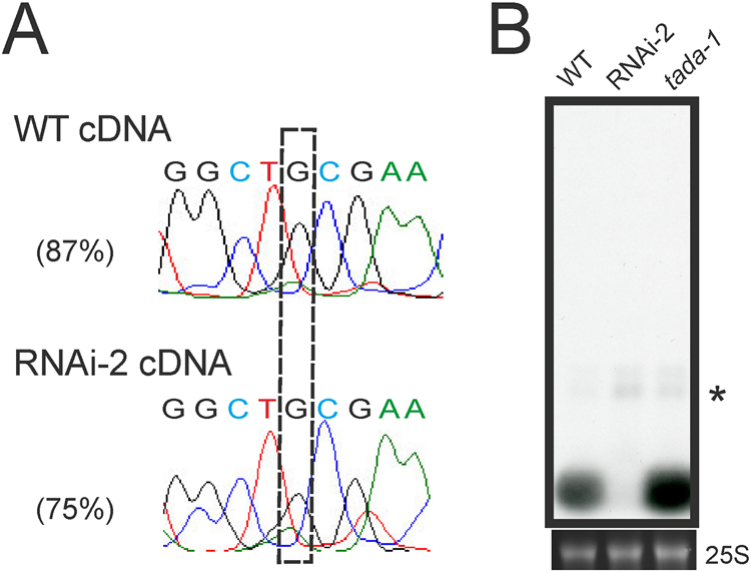
Investigation of the relationship between tRNA editing and 5’ end maturation. (**A**) Analysis of the editing status of tRNA-Arg(ACG) in the chloroplast. The A-to-I editing event changes the adenosine in the wobble position (position 34) of the ACG anticodon to inosine, which is read as guanosine (boxed) by reverse transcriptases [5,6]. Editing efficiencies are shown for the wild type (WT) and the most affected RNAi line (RNAi-2). (**B**) Assessment of the efficiency of tRNA-Arg(ACG) processing by RNase P in the absence of A-to-I editing. *tada-1* is a knock-out allele of the specific adenosine deaminase that edits the anticodon of the plastid tRNA-Arg(ACG) [5,6]. Due to the weak exposure of the blot, the mature tRNA in the RNAi line is hardly visible.

One of the plastid tRNA species that was severely affected in the *PRORP1* mutants is tRNA-Arg(ACG). The abundance of the mature tRNA-Arg(ACG) is strongly reduced in the RNAi-2 line and significant amounts of precursor tRNAs accumulate (that are undetectable in the wild type; Fig. 5A). The plastid tRNA-Arg(ACG) is special in that it is the only plastid tRNA species that undergoes adenosine-to-inosine RNA editing. The editing event changes the adenosine in the wobble position (position 34) of the anticodon to inosine (ACG to ICG). The A-to-I conversion is performed by a dedicated enzyme, an adenosine deaminase (TadA) of which the tRNA-Arg(ACG) is probably the only substrate [5,6]. The editing is functionally important in that it facilitates decoding of CGN arginine codons by wobbling [50]. A-to-I tRNA editing is a post-transcriptional event, but its relationship with tRNA 5’ end processing is unknown. To test whether tRNA 5’ maturation by PRORP1 is required for efficient editing of tRNA-Arg(ACG), the editing status of the tRNA was determined in the wild type and the RNAi-2 mutant (Fig. 8A). While editing in the wild type was nearly complete, it was significantly less efficient in the *PRORP1* RNAi mutant. This could be due to 5’ unprocessed tRNA precursors being less efficient substrates of the TadA editing deaminase. Alternatively, the 5’ unprocessed tRNA molecules could suffer from a higher turnover rate than the mature tRNAs, thus giving the editing enzyme less time to act on them. Further investigations are needed to distinguish between these two possibilities.

Finally, we investigated whether editing at position 34 of tRNA-Arg(ACG) is required for 5’ end processing of the tRNA. To this end, the accumulation of tRNA-Arg(ACG) in the wild type and the RNAi-2 lines was compared to that in a the TadA knock-out mutant *tada-1* [5,6]. Northern blot analyses revealed that mature tRNA-Arg(ACG) accumulates to normal levels in the *tada-1* mutant (Fig. 8B), indicating that 5’ end processing of the tRNA does not depend on prior A-to-I editing.

### Comparative RNAseq reveals additional tRNA processing defects

To obtain a more comprehensive picture of the tRNA processing defects caused by down-regulated PRORP1 activity, we sought to determine the abundances of mature and unprocessed tRNAs by RNA sequencing (RNAseq) using next-generation sequencing technology. High-throughput Illumina sequencing of total RNA samples turned out not to be a suitable approach to analyze tRNA processing. Coverage of mature and unprocessed tRNAs was so low that no statistical analysis could be performed. This is likely due to cDNA synthesis strongly discriminating against tRNAs because of their compact secondary structure (making priming of reverse transcription inefficient) and their extensive post-transcriptional modification with some modifications strongly inhibiting reverse transcription [51]. We, therefore, size-fractionated the RNA samples and constructed libraries enriched for tRNAs (for details, see Materials and Methods).

Illumina sequencing of size-fractionated RNAs yielded sufficient coverage of both mature and unprocessed tRNAs to allow the calculation of processing efficiencies for many, but not all, organellar tRNA species (Tables 1 and 2; S1–S3 Figs.; see also Materials and Methods). The strong underrepresentation of some tRNA species could be due to their low expression levels and/or the presence of RNA modifications that largely block reverse transcription [51].

**Table 1:** Log_2_ ratios of reads mapping to the mature tRNA sequence to reads mapping to the 5’ leader sequence for all tRNAs encoded in the *Arabidopsis* mitochondrial genome.

tRNA	Anticodon	AGI code	Wild type		Log_2_ ratio	RNAi-2		Log_2_ ratio	P value	Adj. P value
			mature	5’l	(ma/5’l)	mature	5’l	(ma/5’l)		
Lys	TTT	ATMG00100 ^3^	493	-	-	475	6	6.31	-	-
Gly	GCC	ATMG00190	169	7	4.59	149	3	5.63	9.18E-001	1.00E+000
Ser	GGA	ATMG00230	15	-	-	69	-	-	-	-
Met_i	CAT	ATMG00250	18	27	-0.58	20	22	-0.14	8.24E-001	1.00E+000
Ser	GCT	ATMG00330	118	1061	-3.17	139	347	-1.32	1.00E+000	1.00E+000
Tyr	GTA	ATMG00340 ^3^	733	85	3.11	292	39	2.90	2.77E-001	1.00E+000
Pro	TGG	ATMG00350	5,618	-	-	5880	1	12.52	-	-
Cys	GCA	ATMG00360	39,297	19	11.01	13,876	-	-	-	-
Asn	GTT	ATMG00380	341	2	7.41	652	4	7.35	6.61E-001	1.00E+000
Tyr	GTA	ATMG00390	154	1	7.27	151	3	5.65	3.09E-001	1.00E+000
Ser	TGA	ATMG00420 ^3^	24	122	-2.35	47	138	-1.55	9.83E-001	1.00E+000
Ile	CAT	ATMG00460	9	3	1.58	10	3	1.74	7.19E-001	1.00E+000
Tyr	GTA	no code ^1^	799	19	5.39	311	27	3.53	2.06-E005	6.58E-004
Ser	GCT	ATMG00780	83	-	-	124	-	-	-	-
Glu	TTC	ATMG00800	267	248	0.11	202	93	1.12	1.00E+000	1.00E+000
Trp	CCA	ATMG00930	1,964	1	10.94	4878	-	-	-	-
Gln	TTG	ATMG00950	45	-	-	83	-	-	-	-
Asp	GTC	ATMG01070	11,777	7	10.72	6608	12	9.11	1.45E-002	4.20E-001
Met_e	CAT	ATMG01340	2	4	-1	-	1	-	-	-
His	GTG	no code ^2^	8,969	-	-	5698	-	-	-	-

^1^Location of tRNA-Tyr(GTA) in the *Arabidopsis* mitochondrial genome is from position 191,954 to 192,025.

^2^Location of tRNA-His(GTG) in the *Arabidopsis* mitochondrial genome is from position 359,666 to 359,739.

^3^The read counts for the two copies of tRNA-Lys(TTT) (ATMG00100 and ATMG00700), the two copies of tRNA-Tyr(GTA) (ATMG00340 and ATMG00790), and the two copies of tRNA-Ser(TGA) (ATMG00420 and ATMG01160) were combined, because both tRNA copies have identical sequences and also identical flanking sequences (up to 50 nt). Dashes indicate transcripts that were not detected in our RNAseq datasets or values that could not be calculated. AGI: Arabidopsis Genome Initiative. 5’l: 5’ leader. ma: mature. Adj. P value: adjusted P value.

**Table 2:** Log_**2**_ ratios of reads mapping to the mature tRNA sequence to reads mapping to the 5’ leader sequence for all intronless tRNAs encoded in the *Arabidopsis* plastid genome.

tRNA	Anti-codon	AGI code	Wild type		Log_2_ ratio	RNAi-2		Log_2_ ratio	P value	Adj. P value
			mature	5’l	(ma/5’l)	mature	5’l	(ma/5’l)		
His	GTG	ATCG00010	587,933	38	13.92	1,039,481	119	13.10	9.37E-004	2.81E-002
Gln	TTG	ATCG00060	10,784	47	7.84	29,908	71	8.72	9.99E-001	1.00E+000
Ser	GCT	ATCG00090	18,967	140	7.08	51,117	170	8.23	1.00E+000	1.00E+000
Arg	TCT	ATCG00110	17,656	60	8.20	25,274	81	8.29	6.68E-001	1.00E+000
Cys	GCA	ATCG00200	402	12	5.07	888	95	3.22	2.45E-006	8.10E-005
Asp	GTC	ATCG00230	423,255	294	10.49	442,408	351	10.30	5.01E-002	1.00E+000
Tyr	GTA	ATCG00240	18,228	135	7.08	11,151	230	5.60	6.05E-022	2.12E-020
Glu	TTC	ATCG00250	73,516	263	8.13	98,975	375	8.04	2.51E-001	1.00E+000
Thr	GGT	ATCG00260	667	232	1.52	1,529	283	2.43	1.00E+000	1.00E+000
Ser	TGA	ATCG00290	136	46	1.56	353	106	1.74	7.56E-001	1.00E+000
Gly	GCC	ATCG00310	106,563	221	8.91	207,724	552	8.56	9.06E-004	2.81E-002
Met_i	CAT	ATCG00320	165,918	1,529	6.76	328,644	2,355	7.12	1.00E+000	1.00E+000
Ser	GGA	ATCG00370	15,326	30	9.00	72,110	49	10.52	9.99E-001	1.00E+000
Thr	TGT	ATCG00390	111	72	0.62	348	149	1.22	9.91E-001	1.00E+000
Phe	GAA	ATCG00410	953	177	2.43	516	536	-0.05	3.71E-071	1.82E-069
Met_e	CAT	ATCG00460	159	37	2.10	406	55	2.88	9.92E-001	1.00E+000
Trp	CCA	ATCG00610	9,553	94	6.67	20,220	108	7.55	9.99E-001	1.00E+000
Pro	TGG	ATCG00620	3,078	55	5.81	9,963	81	6.94	9.99E-001	1.00E+000
Arg	ACG	ATCG00980	4,002	387	3.37	1,000	386	1.37	1.01E-064	3.64E-063
Val	GAC	ATCG00910	945	27	5.13	387	70	2.47	4.29E-017	1.46E-015
Leu	TAG	ATCG01030	6,076	11	9.11	6941	20	8.44	1.43E-001	1.00E+000
Asn	GTT	ATCG01140	7,039	403	4.13	14,784	867	4.09	3.62E-001	1.00E+000
Ala	TGC	ATCG01190	4,763	533	3.16	6,808	309	4.46	1.00E+000	1.00E+000
Leu	CAA	ATCG01260	51,895	119	8.77	97,720	262	8.54	8.61E-002	1.00E+000
Ile	CAT	ATCG01290	97	11	3.14	140	35	2.00	2.04E-002	5.70E-001

AGI: Arabidopsis Genome Initiative. 5’l: 5’ leader. ma: mature. Adj. P value: adjusted P value.

Analysis of the abundance of mature tRNAs confirmed that accumulation of the plastid tRNA-Arg(ACG) and tRNA-Phe(GAA) and the mitochondrial tRNA-Cys(GCA) are strongly reduced in the RNAi line (Tables 1 and 2; S2–S3 Figs). Calculation of a tRNA processing efficiency based on the ratio of mature tRNA reads to precursor reads confirmed the processing defects detected by our northern blot analyses (Fig. 5) and, moreover, revealed additional tRNA species as likely substrates of PRORP1. In the case of the chloroplast, these tRNAs include tRNA-Val(GAC), tRNA-Cys(GCA), tRNA-Tyr(GUA), tRNA-His(GUG) and tRNA-Gly(GCC) (Table 2; S3A Fig.). Due to their much lower copy numbers per cell [52], mitochondrial genes are generally expressed to lower levels than chloroplast genes, and, therefore, the number of mitochondrial tRNA species that gave statistically significant data for processing efficiency was relatively low (Table 1; S3 Fig.). Nonetheless, a tyrosine tRNA, tRNA-Tyr(GUA), could be identified as defective in 5’ end processing in the RNAi-2 mutant and, therefore, is likely to represent an additional substrate of PRORP1 (Table 1; S3C Fig.).

In several cases, the reduced accumulation of mature tRNA molecules was also accompanied with overrepresentation of 3’ unprocessed tRNAs (S3B,D Fig.). This was the case, for example, for the two most strongly reduced plastid tRNAs, tRNA-Arg(ACG) and tRNA-Phe(GAA) (S3B Fig.). It seems likely that this is a secondary consequence of inefficient 5’ processing, but due to the short reads generated by the Illumina sequencing technology, we currently do not know whether (most of) these 3’ unprocessed molecules are also 5’ unprocessed.

## Conclusions

Our reverse genetic analysis of the dually targeted proteinaceous RNase P revealed that the effects of knocked-down *PRORP1* on the processing of individual tRNA species in chloroplasts and mitochondria are highly variable. While a few tRNAs are severely affected, many others show little or no changes in accumulation of the mature tRNA. This might suggests that the organellar RNase P recognizes some tRNA substrates more efficiently than others. The molecular basis for these substrate preferences is currently unclear. Comparison of sequences upstream of tRNA 5’ ends failed to reveal recognizable motifs that could correlate with processing efficiency (data not shown), raising the possibility that higher-order structure of the tRNA precursors influences their recognition by PRORP1. It is noteworthy in this respect that the mitochondrial tRNA most affected by knock-down of *PRORP1*, tRNA-Cys(GCA) (Fig. 5B), has a somewhat unusual secondary structure and contains an C⋅A mismatch in a stem region which is not corrected to a U⋅A base pair by RNA editing [53]. Alternatively, it seems possible that differences in the tRNA turnover rates (i.e., the balance between synthesis and degradation) and/or the interplay with other tRNA maturation steps are, at least in part, responsible for the observed large differences in the effect of *PRORP1* knock-down on the processing of individual tRNA species. Finally, it also cannot be excluded that another RNase P-like activity exists in plant organelles that is distinct from the PRORP protein family and yet remains to be discovered.

The processing defects reported here in stable transgenic RNAi mutants are similar but not absolutely identical to the defects described previously upon transient repression of *PRORP1* gene expression using virus-induced gene silencing [8]. For example, Gutmann et al. observed less mature mitochondrial tRNA-Lys(UUU), which is not supported by our RNAseq data (Table 1). However, consistent with the previous study by Gutmann et al., 5’ unprocessed precursors of mitochondrial tRNA-Lys(UUU) are detectable in the RNAi line but not in the wild type (Table 1), suggesting that mitochondrial tRNA-Lys(UUU) is indeed a substrate of PRORP1. Another difference between the two studies is that the reduction in mature chloroplast tRNA-Phe(GAA) is stronger in our stable RNAi plants. Whether or not these differences are solely due to differences in the level of *PRORP1* repression or have other reasons, remains to be determined by future investigations.

The physiological analysis of our *PRORP1* mutants indicated that photosynthesis (as the main function of plastid gene expression) is much more strongly affected than respiration (the main function dependent on mitochondrial gene expression), suggesting that the mutant phenotype is largely caused by impaired chloroplast gene expression. Moreover, the drastic reduction in mature plastid tRNA-Phe(GAA) and tRNA-Arg(ACG) suggests these two tRNA species as a major limiting factor in plastid gene expression in *PRORP1* mutants. Although plant mitochondria, unlike chloroplasts, import some tRNAs from the cytosol (one third to half of the plant mitochondrial tRNA species are encoded in the nucleus), tRNA import cannot explain the lack of effects on mitochondrial function and gene expression in our RNAi mutants, because tRNA import is restricted to a specific set of tRNA species [54] which can be identified by their nucleotide sequences [55].

## Supporting Information

S1 Fig Read annotation statistics for the RNAseq experiment. Genome mapped reads were annotated according to their first assignment to a genomic feature using intersectBed in the following order: exons, 5’ and 3’ untranslated regions (‘prime UTRs’), repeats (excluding 'dust') and tRNA flanks (±50 bp of mature tRNA locations). See Materials and Methods for details.
(TIF)

S2 Fig MA plot of the accumulation of mature tRNAs in wild-type plants and RNAi-2 mutant plants. Expression of mature tRNAs was quantified by computing RPKM values for unique tRNA sequences which were used to calculate differences (M) and averages (A). Density plots show the distribution of M and A, respectively. The M values on the vertical axis represent differential accumulation between the wild type and the RNAi line. The A values on the horizontal axis represent average tRNA accumulation levels. Red dots: mitochondrial genome-encoded tRNAs; green dots: plastid-encoded tRNAs; gray dots: nucleus-encoded tRNAs. Experimentally validated tRNAs are highlighted by their three-letter amino acid code.
(TIF)

S3 Fig MA plots of the efficiency of tRNA processing in wild-type plants and RNAi-2 mutant plants. A processing efficiency rate (PER), defined as the log_2_ ratio of reads mapped on mature and 3’ or 5’ flanking region, respectively, was computed for each tRNA in the wild type and the RNAi line to calculate differences (M) and averages (A). High PER values indicate efficient tRNA processing. Negative M values indicate a higher PER in the wild type than in the RNAi line. P values were computed in R using the Fisher exact test with Benjamini-Hochberg multiple testing correction based on read counts used for PER calculation. (**A**) PERs for 5’ regions of chloroplast tRNAs. (**B**) PERs for 3’ regions of chloroplast tRNAs. (**C**) PERs for 5’ regions of mitochondrial tRNAs. (**D**) PERs for 3’ regions of mitochondrial tRNAs. tRNA species are indicated by the three-letter amino acid codes. Green: experimentally tested chloroplast tRNAs; slate blue: experimentally validated chloroplast tRNAs with adjusted P value < 0.05; red: experimentally tested mitochondrial tRNAs; gray: not experimentally tested chloroplast or mitochondrial tRNAs; black: not experimentally tested chloroplast or mitochondrial tRNAs with adjusted P value < 0.05.
(TIF)

S4 Fig Weaker exposures of the northern blots for tRNA-Glu(UUC), tRNA-Gln(UUG) and tRNA-Asp(GUC) shown in Fig. 5. (TIF)

S1 Table Gene-specific primers used for the generation of hybridization probes. (DOC)
