# Supplementary figures and images for: Multiple RNA Processing Defects and Impaired Chloroplast Function in Plants Deficient in the Organellar Protein-Only RNase P Enzyme
Source: PLoS One. 2015 Mar 20;10(3):e0120533. doi: 10.1371/journal.pone.0120533 (PMC4368725; doi:10.1371/journal.pone.0120533)

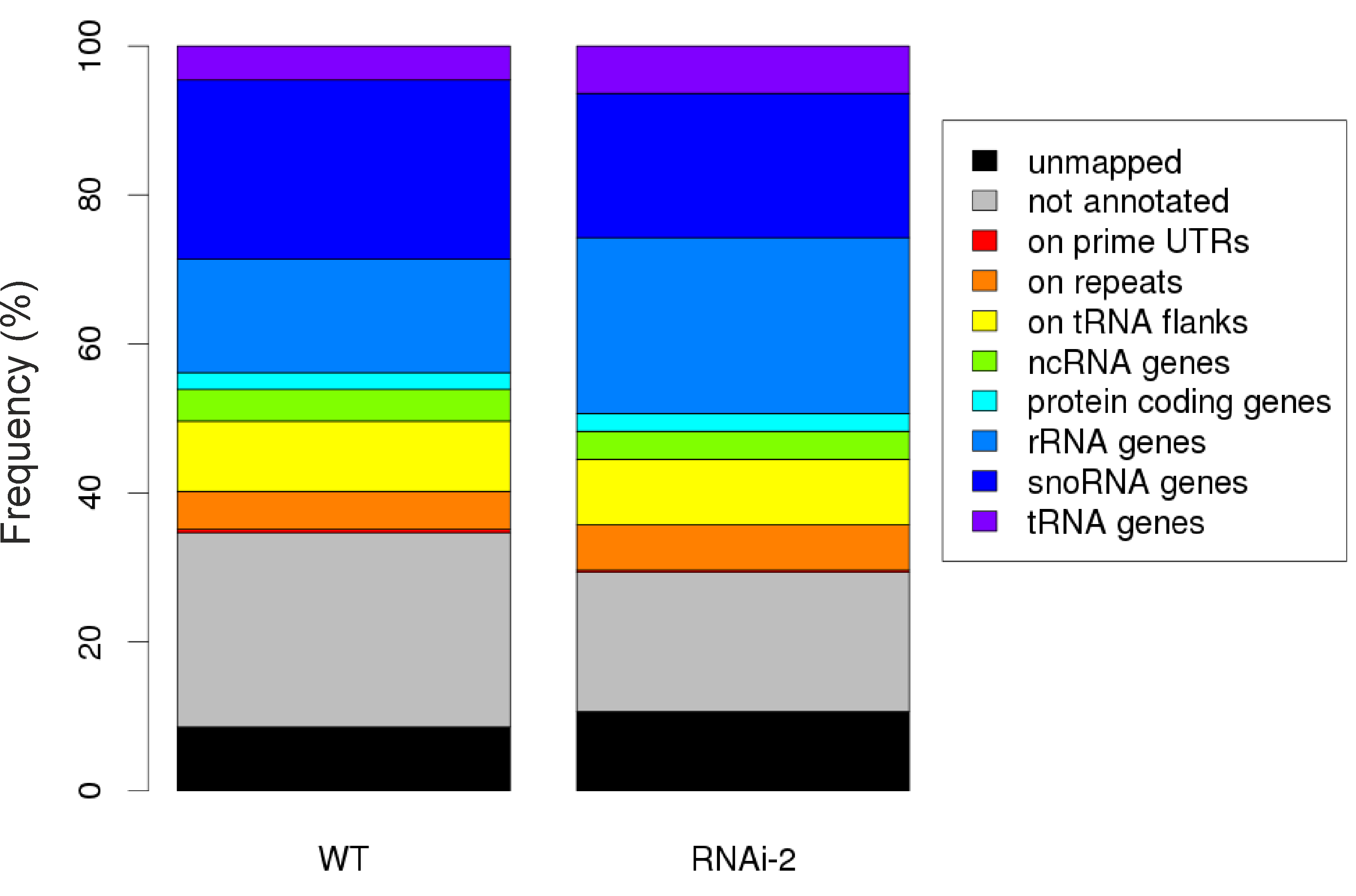

Supplement: S1 Fig — Genome mapped reads were annotated according to their first assignment to a genomic feature using intersectBed in the following order: exons, 5’ and 3’ untranslated regions (‘prime UTRs’), repeats (excluding 'dust') and tRNA flanks (±50 bp of mature tRNA locations). See Materials and Methods for details. (TIF) [file pone.0120533.s001.tif]

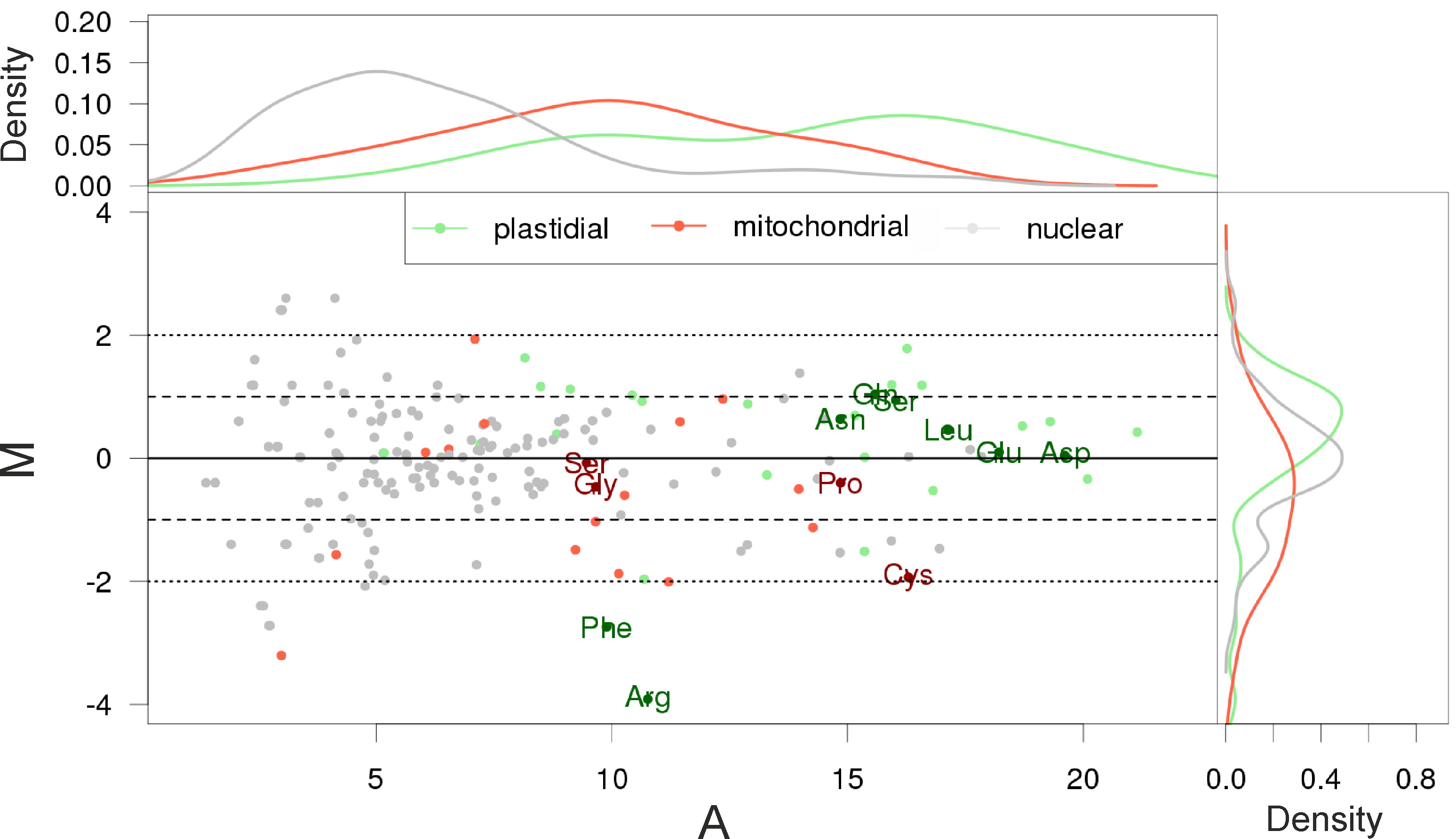

Supplement: S2 Fig — Expression of mature tRNAs was quantified by computing RPKM values for unique tRNA sequences which were used to calculate differences (M) and averages (A). Density plots show the distribution of M and A, respectively. The M values on the vertical axis represent differential accumulation between the wild type and the RNAi line. The A values on the horizontal axis represent average tRNA accumulation levels. Red dots: mitochondrial genome-encoded tRNAs; green dots: plastid-encoded tRNAs; gray dots: nucleus-encoded tRNAs. Experimentally validated tRNAs are highlighted by their three-letter amino acid code. (TIF) [file pone.0120533.s002.tif]

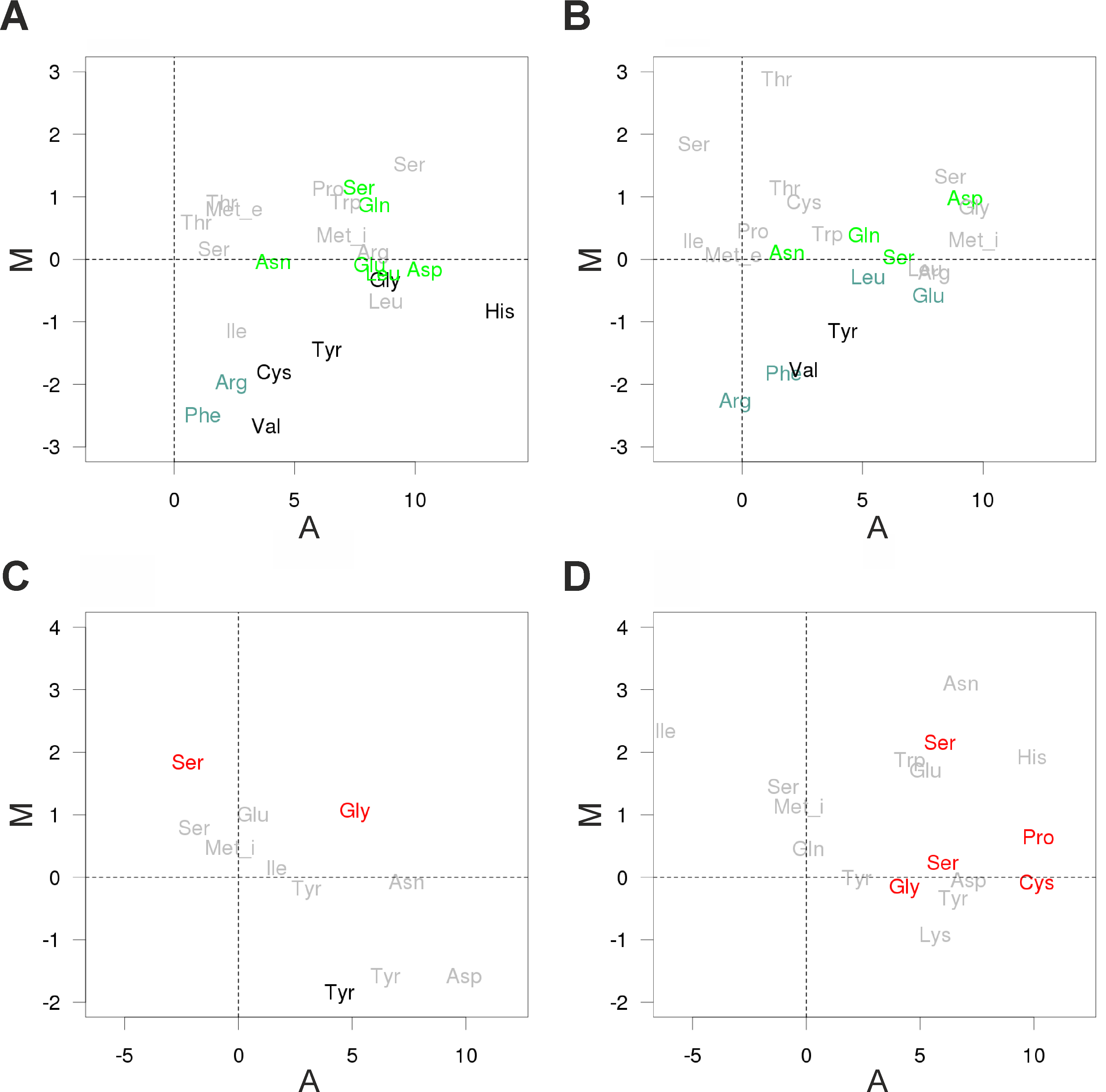

Supplement: S3 Fig — A processing efficiency rate (PER), defined as the log2 ratio of reads mapped on mature and 3’ or 5’ flanking region, respectively, was computed for each tRNA in the wild type and the RNAi line to calculate differences (M) and averages (A). High PER values indicate efficient tRNA processing. Negative M values indicate a higher PER in the wild type than in the RNAi line. P values were computed in R using the Fisher exact test with Benjamini-Hochberg multiple testing correction based on read counts used for PER calculation. (A) PERs for 5’ regions of chloroplast tRNAs. (B) PERs for 3’ regions of chloroplast tRNAs. (C) PERs for 5’ regions of mitochondrial tRNAs. (D) PERs for 3’ regions of mitochondrial tRNAs. tRNA species are indicated by the three-letter amino acid codes. Green: experimentally tested chloroplast tRNAs; slate blue: experimentally validated chloroplast tRNAs with adjusted P value < 0.05; red: experimentally tested mitochondrial tRNAs; gray: not experimentally tested chloroplast or mitochondrial tRNAs; black: not experimentally tested chloroplast or mitochondrial tRNAs with adjusted P value < 0.05. (TIF) [file pone.0120533.s003.tif]

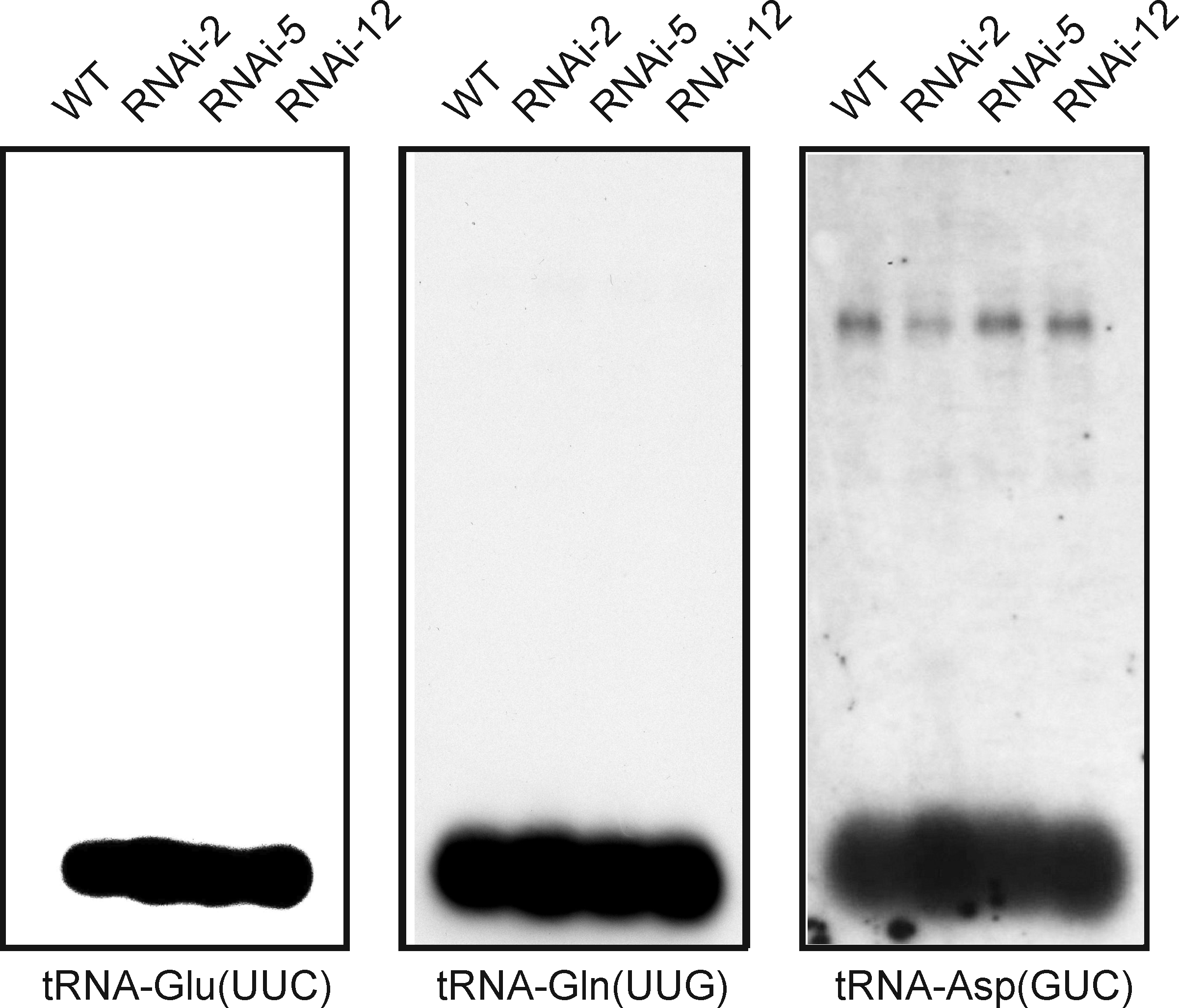

Supplement: S4 Fig — (TIF) [file pone.0120533.s004.tif]
