# Supplementary material for: Multiple RNA Processing Defects and Impaired Chloroplast Function in Plants Deficient in the Organellar Protein-Only RNase P Enzyme
Source: PLoS One. 2015 Mar 20;10(3):e0120533. doi: 10.1371/journal.pone.0120533 (PMC4368725; doi:10.1371/journal.pone.0120533)
Supplement: S1 Table — (DOC) [file pone.0120533.s005.doc]

**S1 Table.** Gene-specific primers used for the generation of hybridization probes.

| **Gene** | **Forward primer sequence 5´→3´** | **Reverse primer sequence 5´→ 3´** |
| --- | --- | --- |
| Chloroplast genes | | |
| *psaA* | CAATTGGCGCATTGGTCTTCGCAG | GTGCTCGCTGTTTCACCAGGGGCTG |
| *psbA* | ATGACTGCAATTTTAGAGAGACGCG | TTATCCATTTGTAGATGGAGCCTCA |
| *petB* | TATGATTGGTTCGAAGAACGTCTTG | TTATAAGGGACCAGAAATACCTTGC |
| *atpB* | GAGCGACTCTAGGACGAATTTTC | TCTAAATGTGCAAATGTGGTGGC |
| *trnR(ACG)* | GGGCTTGTAGCTCAGAGG | TGGTGGGCGAGGAGGGATTC |
| *trnD(GUC)* | GGGATTGTAGTTCAATTGGTCAGAGC | CGGGACTGACGGGGCTCGAAC |
| *trnE(UUC)* | GCCCCCATCGTCTAGTGGTTCAG | TACCCCCAGGGGAAGTCGAATC |
| *trnQ(ACG)* | TGGGGCGTAGCCAAGCGGTAAG | CTGGGACGGAAGGATTCGAACC |
| *trnS(GCU)* | CGGAAAGAGAGGGATTCGAA | GGAGAGATGGCTGAGTGGAC |
| *trnL(CAA)* | TGCCTTGAAGAGGACTCG | GCCTTGGTGGTGAAATGGTAG |
| *trnF(GAA)* | GCCGGGATAGCTCAGTTGGTA | TGCCAAGAACCAGATTTGAAC |
| *trnN(GUU)* | TCCTCAGTAGCTCAGTGGTAGA | CTCCCCAAGTAGGATTCGAACC |
| *rrn23* | TTCAAACGAGGAAAGGCTTACGGTG | AGGAGAGCACTCATCTTGGGGTGG |
| *rrn16* | tctcatggagagttcgatcctg | cttatcaccggcagtctgttc |
| *rrn5* | TATTCTGGTGTCCTAGGCGTAG | TTTCCGCAGGACCTCCCCTACA |
| *rrn4.5* | GACGAGCCGTTTATCATTACGATAG | TTCAAGTCTACCGGTCTGTTAGG |
| Mitochondrial genes | | |
| *nad2* | TACGCTTAGTGAAAAGAATG | ATCGAGCACCAGTGATTTCGTATC |
| *nad6* | ATGATACTTTCTGTTTTGTCGAGC | CGTGAGTGGGTCAGTCGTCC |
| *cob* | GTAGGAGATACCATAGTGAC | CTGATCAGGTGTGATCAGTC |
| *cox1* | CTTAGACGTAGATACCCGTG | CATAGCTTTTCGTCTCCTTG |
| *cox2* | TTCTCCTTGTGATGCAGCGG | CTCACTGCACTGACCATAGT |
| *trnC(GCA)* | GGCTAGGTAACATAATGGAAATGTATC | GCTTTCCCTTTGTTCCAGTTATTTC |
| *trnG(GCC)* | TGGCGGAAATAGCTTAATGGTAGAGC | AGCGGAAGGAGGGACTTGAACCC |
| *trnS(GCU)* | GGAGGGATGGCTGAGTGGCTTAAG | CGGAGGAAATGGGATTCGAAC |
| *trnP(UGG)* | CGAGGTGTAGCGCAGTCTGGTC | TCAAGGTGACAGGATTCGAACC |
